# From Environmental Burden to Structural Alterations: Integrating Chemical Analysis and Fluorescence Spectroscopy in European Chub (*Squalius cephalus*)

**DOI:** 10.3390/toxics14070580

**Published:** 2026-06-30

**Authors:** Dušan Nikolić, Mira Stanković, Dragana Bartolić, Danica Rilak, Ksenija Radotić

**Affiliations:** 1 Department of Biodiversity and Environmental Research, University of Belgrade—Institute for Multidisciplinary Research, National Institute of the Republic of Serbia, Kneza Višeslava 1, 11030 Belgrade, Serbia; 2 Department of Life Sciences, University of Belgrade—Institute for Multidisciplinary Research, National Institute of the Republic of Serbia, Kneza Višeslava 1, 11030 Belgrade, Serbia; mira.mutavdzic@imsi.rs (M.S.); dragana.bartolic@imsi.rs (D.B.); xenia@imsi.bg.ac.rs (K.R.); 3 Faculty of Agriculture, University of Belgrade, Nemanjina 6, Zemun, 11080 Belgrade, Serbia; danicarilak10@gmail.com

**Keywords:** fish, fish meat, potentially toxic elements, aquatic pollution, biomonitoring, fluorescence spectroscopy

## Abstract

Freshwater fish are important bioindicators of environmental pollution and provide a pathway for human exposure to toxic elements. We aimed to determine the concentrations of 29 elements in the muscle tissue of European chub collected from the Pek River (impacted by mining activities and untreated wastewater), the Ibar River (affected by treated wastewater), and the Kruščica Reservoir (an unpolluted drinking water source). Elemental analysis was conducted using ICP-OES, and the results were compared to maximum allowed concentrations (MACs) for fish meat, alongside a human health risk assessment. No significant spatial differences were observed for most elements; elevated Pb levels were found in several individuals from Kruščica and Ibar, while isolated cases of increased Cd were recorded at all sites. Fluorescence spectroscopy was used to assess structural changes in muscle tissue, particularly in collagen. A red shift in collagen emission maxima was observed along the pollution gradient (Kruščica < Ibar < Pek), indicating structural modifications. These findings are consistent with the elevated metal burden detected in Pek. Overall, despite localized contamination, consumption of chub appears safe. The strong correlation between elemental composition and fluorescence responses demonstrated the potential of fluorescence spectroscopy as a rapid tool for detecting pollution-induced tissue alterations.

## 1. Introduction

Fish are of great importance both ecologically and nutritionally. Within aquatic ecosystems, they contribute to biodiversity, regulate trophic dynamics, participate in nutrient cycling, and serve as reliable indicators of environmental health. From a dietary perspective, fish meat is a valuable source of high-quality protein and essential nutrients, playing a significant role in human nutrition worldwide [1]. Compared to other tissues, muscle has the lowest affinity for element accumulation [2,3,4]. Nevertheless, it is frequently analyzed in ecotoxicological studies, primarily because it represents the edible portion of fish and is directly relevant to human consumption [5,6,7].

Fluorescence spectroscopy has emerged as a powerful, rapid, and non-destructive analytical tool for monitoring the quality of fish muscle [8,9]. It has been applied to the assessment of pollutant exposure in aquatic organisms, including fish tissues [10], where fluorescent biomarkers reflect environmental contamination and organismal uptake of pollutants. This technique enables sensitive detection of intrinsic fluorophores such as collagen [8], whose fluorescence properties change in response to physiological stress and contaminant exposure. Variations in fluorescence emission spectra can indicate alterations in protein conformation, oxidative status, and metabolic activity, providing valuable insights into tissue degradation processes, and sublethal toxic effects.

The European chub, *Squalius cephalus* (Linnaeus, 1758), inhabits the basins of the North, Baltic, White, and Barents Seas, as well as the Caspian Sea (Volga and Ural Rivers), the Black Sea (Danube and Dniester Rivers), the Mediterranean Sea (Var and Ebro Rivers), and the Atlantic Ocean basin [11,12]. It has also been introduced into the waters of Italy and Ireland. In Serbia, the species occurs in both lotic and lentic ecosystems, including numerous reservoirs where it has successfully acclimatized. It typically prefers rivers and streams with faster flow (the rhithral zone), but readily adapts to lake-like conditions in reservoirs regardless of altitude. It can also be found in slow-flowing lowland rivers and tolerates both freshwater and brackish habitats [12]. This species is an omnivorous and opportunistic feeder. Juveniles primarily consume plankton and various aquatic invertebrates, while adults shift towards a more diverse diet that includes plant material and fish. Although precise population trends are unknown, the species is widespread and abundant, suggesting stable populations. The chub has limited economic importance in commercial fisheries. However, it is highly valued and popular in recreational angling [12]. For these reasons, this species is frequently used as a model organism in ecotoxicological research [3,4,7,13,14,15].

This study aimed to determine the concentrations of chemically heterogeneous elements (Ba, Ca, K, Li, Mg, Na, Sr, Ag, Cd, Co, Cr, Cu, Fe, Mn, Mo, Ni, Pt, Rh, Ti, Zn, Al, Pb, Sn, As, B, Sb, P, S, and Se) in the muscle tissue of the European chub collected from the Pek River (subjected to long-term mining activities and untreated municipal wastewater), the Ibar River (affected by treated municipal wastewater discharge), and the Kruščica Reservoir (a drinking water supply). In ecotoxicological studies, particular emphasis is generally placed on elements commonly classified as heavy metals due to their persistence, bioaccumulation potential, and toxic effects on aquatic organisms [16]. However, the inclusion of macroelements and essential trace elements in this study enabled a more comprehensive assessment of elemental profiles, environmental exposure, and physiological responses across different tissues. Furthermore, the broad elemental dataset generated may serve as a valuable reference framework for future comparative investigations and contribute to the standardization and interpretation of ecotoxicological studies in aquatic ecosystems. The results were further compared with fluorescence spectroscopy parameters to monitor pollution-induced structural alterations in fish muscle and to assess whether fluorescence spectroscopy reflects the elemental burden detected in the muscle tissue, as well as to evaluate its potential as a rapid screening method for detecting structural changes caused by environmental pollution. To our knowledge, this is the first study in which fluorescence spectroscopy has been applied to the analysis of muscle tissue in wild freshwater fish. The novelty of this work also lies in the combined interpretation of elemental burdens and fluorescence signatures in fish muscle tissue collected from ecosystems exposed to varying levels of anthropogenic pressure.

The general hypothesis was that sites under greater anthropogenic pressure, specifically the Pek River and the Ibar River, would exhibit higher accumulation of certain elements and a greater metal pollution burden along with altered fluorescence patterns, compared to the Kruščica Reservoir. The rationale is that results from this non-destructive, rapid fluorescence analysis can serve as a preliminary assessment, helping determine whether selected samples warrant further investigation using complementary, more precise analytical techniques.

## 2. Materials and Methods

### 2.1. Study Area

**Figure 1:**
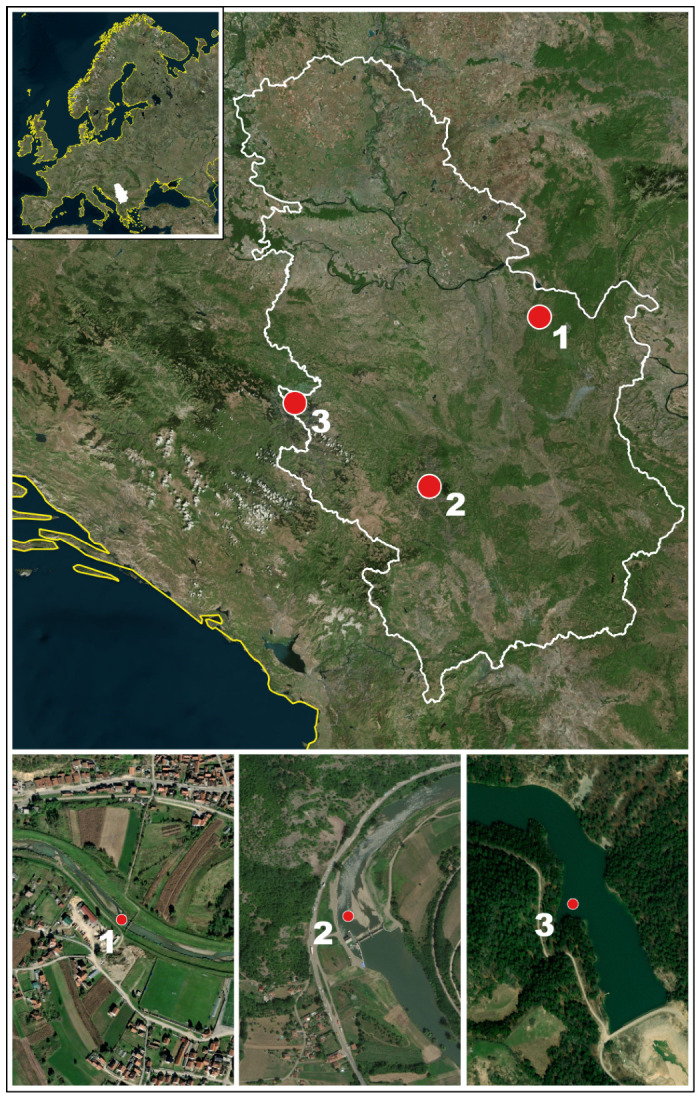
Map of the sampling sites: (1) Pek River; (2) Ibar River; (3) Kruščica Reservoir.

Fish were sampled between October 2021 and March 2022 at three freshwater sites in Serbia (Figure 1):

1. The Pek River (44°28′35.6″ N, 21°39′40.5″ E; 148 m above sea level) is a 129 km long right-bank tributary of the Danube in eastern Serbia. The main sources of pollution in the Pek River basin include the ″Majdanpek″ copper mine, a copper pipe factory and the ″Kaona″ quarry, all situated directly along the river. Elevated concentrations of copper, sulphates, nitrogen, ammonia, orthophosphates, iron, and manganese have been recorded in both water and sediment. Prolonged mining activities, which have historically been the dominant economic sector in this region, have resulted in severe contamination of downstream waters with toxic elements and sulphates originating from the mining area [17].
2. The Ibar River (43°20′20.5″ N, 20°38′10.7″ E; 391 m above sea level) is located in the southern and central parts of Serbia, with a total length of 276 km and a drainage area of 8059 km^2^. For most of its course, it flows between mountains (the Ibar Gorge). The water of the Ibar River is a typical example of a river that can be used for hydroelectric power development, eco-tourism zones, and the promotion of sustainable development in rural areas. However, its potential for economic development remains insufficiently utilized. In its middle and lower courses, the Ibar River receives large amounts of wastewater from various sources. Some of this wastewater originates from industry, some from agriculture, some from numerous industrial and municipal landfills in the area, and some from untreated sanitary and faecal waters that are discharged directly [18].
3. The Kruščica Reservoir (43°54′06.1″ N, 19°23′05.1″ E; 900 m above sea level) is located in western Serbia, within the territory of “Tara” National Park. It is protected from anthropogenic impact and is used to supply water to the local population [19]. The reservoir is situated on the western side of Zaovine Reservoir and is surrounded by forest and a pristine natural environment.

### 2.2. Fish Sampling and Sample Preparation

European chub individuals from the Ibar and Pek rivers were sampled by the electrofishing device Villager VGI2400 (230 V, 8.7 A, 2.0 kW; Villager d.o.o., Ljubljana, Slovenia), while specimens from the Kruščica Reservoir were collected using cylindrical fyke nets with two conical funnel entrances (85 × 50 cm, 8 mm mesh size). Each individual was measured, total length (TL, cm) and weight (W, g). Age was determined by examining growth zones on scales, and sex was identified through macroscopic examination of the gonads.

Samples of the left dorsal muscle, taken beneath the dorsal fin for elemental concentration analysis, were rinsed with distilled water and stored at −20 °C until further processing. Sample mass was recorded before and after freeze-drying using a rotational vacuum freeze dryer (GAMMA 1–16 LSC, Martin Christ Gefriertrocknungsanlagen GmbH, Osterode am Harz, Germany). Portions of the dried tissue (0.12–0.50 g) were digested in a microwave digestion system (ETHOS EASY, Milestone, Sorisole (Bergamo) Italy) using the Dried Fish program (200 °C), with the addition of 6 mL of 65% nitric acid and 4 mL of 30% suprapure hydrogen peroxide (Merck KGaA, Darmstadt, Germany). The resulting digests were diluted with distilled water to 25 mL prior to elemental analysis.

Fresh fish muscle tissues were used for analysis and kept on ice prior to measurements to preserve their native properties. A representative portion of muscle tissue was aseptically excised, and fluorescence spectra were acquired directly from the sample surface using an optical fiber probe. Spectral measurements were conducted under controlled conditions to minimize external interference and ensure reproducibility.

### 2.3. Element Analysis

Element concentrations, expressed as μg/g dry weight (dw), were determined by inductively coupled plasma optical emission spectrometry (ICP-OES, Avio 200, PerkinElmer Inc., Waltham, MA, USA). Concentrations of 29 selected chemical elements with diverse chemical properties were determined, including alkali and alkaline earth metals (Ba, Ca, K, Li, Mg, Na, Sr), transition metals (Ag, Cd, Co, Cr, Cu, Fe, Mn, Mo, Ni, Pt, Rh, Ti, Zn), post-transition metals (Al, Pb, Sn), metalloids (As, B, Sb), and non-metals (P, S, Se). Elemental determinations were performed at the following analytical wavelengths (λ, nm): Ba (233.527), Ca (317.933), K (766.490), Li (670.784), Mg (285.213), Na (589.592), Sr (407.771), Ag (328.068), Cd (228.802), Co (228.616), Cr (267.716), Cu (327.393), Fe (238.204), Mn (257.610), Mo (202.031), Ni (231.604), Pt (265.945), Rh (343.489), Ti (334.940), Zn (206.200), Al (396.153), Pb (220.353), Sn (189.927), As (193.696), B (249.677), Sb (206.836), P (213.617), S (181.975), and Se (196.026).

To assess potential contamination from reagents, six analytical blank samples without muscle tissue were processed using the same procedure as the muscle tissue samples. Quality control of the analytical procedure was ensured through the analysis of certified reference materials, BCR-185R Bovine Liver (European Commission Joint Research Centre) and IAEA-336 Lichen (AQCS, International Atomic Energy Agency). Multi-element standard solutions were used to construct calibration curves covering the anticipated concentration ranges of the analyzed samples. A minimum of five calibration levels and blanks were used for each of the 29 elements analyzed in this study. Assessment of calibration linearity was based on visual inspection of correlation coefficients, calibration plots, residual errors, and residual distributions across all calibration levels. We accepted calibration ranges in the following cases: (1) linear response was observed; (2) residual errors remained within acceptable limits particularly at concentration levels corresponding to the analyzed samples. For all elements analyzed, limits of detection (LOD), limits of quantification (LOQ), and recovery values are presented in Table S1. When element concentrations were below the instrumental limits of detection, values corresponding to one-half of the ICP-OES detection limit were used for statistical analysis.

Element concentrations in muscle tissue (μg/g wet weight) were compared with the prescribed maximum allowed concentrations (MACs) for fish meat in accordance with the national legislation of the Republic of Serbia [20] and the European Commission regulation [21]. These regulations are harmonized with respect to the MACs for Cd (0.05 μg/g ww) and Pb (0.3 μg/g ww). In addition, the national legislation of the Republic of Serbia specifies MACs for Cu (30 μg/g ww), Zn (100 μg/g ww), and As (2 μg/g ww).

To assess the overall toxicity of the samples based on potentially toxic metals (μg/g ww), the Metal Pollution Index (MPI) was calculated using the following formula [22]:

(1) $$MPI={\left(C_{1}\times C_{2}\times \ldots \;\times C_{n}\right)}^{1/n}$$

where *C_n_* represents the concentration of the *n*th metal in a given tissue, expressed in μg/g ww.

### 2.4. Fluorescence Spectroscopy of Muscle Tissue

Fluorescence measurements were performed using a spectrofluorometer (FL3-221, Horiba, Paris, France) equipped with a 450 W high-pressure xenon lamp and a quartz optical fibre probe (effective diameter 4 mm) positioned in a right-angle configuration at a distance of 2 mm from the sample surface. Emission spectra in the range 360–525 nm were recorded directly from muscle tissue surfaces upon excitation at 325 nm. The excitation and emission slit widths were both set to 0.70 nm, with a step size of 1 nm and an integration time of 0.1 s. All measurements were performed in triplicate for each sample at room temperature under dark conditions. Spectral acquisition and data processing were carried out using FluorEssence 3.5 software (Horiba Scientific, Kyoto, Japan). The spectra were normalized for further comparisons.

### 2.5. Health Risk Assessment

The health risk assessment was conducted based on the following assumptions:

1. The cooking process does not affect contaminant concentrations;
2. The ingested dose of contaminants is assumed to be equal to the absorbed dose;
3. An average food ingestion rate (FIR) of 20 g/d was applied for European chub consumption [23];
4. The average body weight (BWa) is assumed to be 63 kg for women and 83 kg for men [24];
5. The average life expectancy in Serbia is 78.1 years for women and 73.2 years for men [25];
6. Mean and maximum concentrations of each element were applied in the human health risk assessment;
7. Inorganic As accounts for 3% of the total As content [26].

#### 2.5.1. Target Hazard Quotient (THQ)

The target hazard quotient (THQ) was estimated using the following formula [27]:

(2) $$THQ=((EFr\times ED\times FIR\times C)/(BWa\times AT\times RfD))\times 10^{-3}$$

The parameter EFr represents the exposure frequency and was set at 365 days per year; ED denotes the exposure duration and corresponds to the average life expectancy of men and women; C indicates the concentration of a given element in fish muscle (μg/g). AT is the averaging time for non-carcinogenic substances and is calculated 365 days per year multiplied by the number of exposure years. RfD denotes the oral reference dose (mg/kg/d), with values of 0.0001 for Cd, 1.5 for Cr, 0.04 for Cu, 0.3 for Zn, 0.004 for Pb, and 0.0003 for As [28].

The total target hazard quotient (TTHQ) is expressed as the sum of all calculated THQ values:

(3) TTHQ=∑THQ

#### 2.5.2. Target Carcinogenic Risk Factor (TR)

The formula used to calculate this index, referred to as the target carcinogenic risk factor (TR), is as follows [27]:

(4) $$TR=((EFr\times ED\times FIR\times C\times {CSF}_{O})/(BWa\times AT))\times 10^{-3}$$

The difference compared to the THQ lies in the CSFo parameter, which represents the oral carcinogenic slope factor (mg/kg/d) and has values of 0.0085 for Pb and 1.5 for As. AT denotes the averaging time for carcinogenic substances and is calculated as 365 days per year multiplied by the exposure duration (ED) [26]. A cancer risk is considered negligible when TR < 10^−6^, while values in the range 10^−6^ < TR < 10^−4^ are regarded as tolerable [27].

### 2.6. Statistical Analysis

Statistical analyses were conducted using STATISTICA software (Version 8.0, StatSoft Inc., Tulsa, OK, USA). A probability level of *p* ≤ 0.05 was considered statistically significant for all analyses. Results are presented as mean values ± standard deviation (SD). When element concentrations were below the detection limit (not detected, ND), values equal to one half of the element-specific detection limit of the ICP-OES spectrometer were used (Table S1). Data normality was assessed using the Shapiro–Wilk test. For normally distributed data, homogeneity of variances was evaluated with Levene’s test. Subsequently, one-way analysis of variance (ANOVA) followed by Tukey’s HSD post hoc test was applied to determine statistically significant differences. When data did not meet the assumptions of normality, differences among sampling sites were assessed using the Kruskal–Wallis *H* test, with pairwise comparisons performed using the non-parametric Mann–Whitney *U* test. The fluorescence data were normalized and standardized. Subsequently, principal component analysis (PCA) and correlation matrix analysis were performed using XLSTAT 2018 software (Addinsoft Inc., New York, NY, USA). Differences among groups were assessed using permutational multivariate analysis of variance (PERMANOVA) based on Euclidean distance matrices with 999 permutations.

## 3. Results

### 3.1. Length and Weight of the Sampled Individuals

The largest average length and weight, as well as the highest condition factor values, were recorded for individuals from the Ibar River. In contrast, the smallest average body length and weight, as well as the lowest condition factor value, were recorded for fish from the Pek River (Table 1). However, the differences in condition factor values were not statistically significant. All analyzed individuals were juveniles, with the majority belonging to the 1+ age class. All specimens appeared clinically healthy, were in good overall condition, and showed no visible signs of injury, lesions, or external abnormalities.

### 3.2. Element Concentrations

The highest concentrations of Pb, Zn, P, K, Na and Ni in muscle tissue were recorded at the Kruščica Reservoir. On the other hand, the lowest concentrations of Ba, Sr and Ca were recorded at the Ibar River (Table 1). B, Co, Li, Rh and Mn were not detected in any sampled individuals at any locality. There were no significant differences between sampling localities regarding the concentrations of Al, As, Cd, Cr, Cu, Ag, Pt, Ti, Fe, and Mg. No statistically significant differences were found among the analyzed elements in relation to the age and size of the examined fish.

Concentrations of As. Cu. and Zn in muscle tissue did not exceed the MAC values prescribed by national or European Commission regulations. However, concentrations of Pb and Cd exceeded the prescribed MACs in sampled individuals from all three localities. For Pb, elevated values were recorded in two individuals from the Kruščica Reservoir and three individuals from the Ibar River, while one individual from each of the three localities had elevated Cd values.

According to the obtained MPI values, the highest metal load was recorded in the Pek River (Table 1).

### 3.3. Fluorescence Spectroscopy of Muscle Tissue

**Figure 2:**
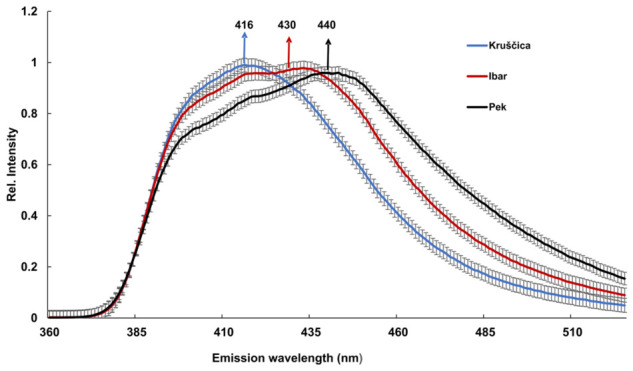
Averaged fluorescence emission spectra of the muscles from the fish collected at the three localities. Emission spectra represent mean values of independent samples from each location (8 for Kruščica and 10 for Ibar and Pek). Shaded areas indicate the 95% confidence intervals at each wavelength. The arrows indicate positions of the emission maxima. Excitation wavelength: 325 nm.

The fluorescence spectra of the muscles from Ibar and Pek have a similar shape which differs from the Kruščica samples (Figure 2). The spectral maxima of the muscles from Kruščica, Ibar, and Pek are 416, 430 and 440 nm, respectively. Thus, the maxima for Ibar and Pek are shifted by 14 nm and 24 nm, respectively, towards higher wavelengths compared to that from Kruščica. Additionally, the maximum for Pek is red-shifted by 10 nm compared to that of Ibar. Statistical comparison using PERMANOVA has shown significant differences in emission maxima positions between sampling locations (Pseudo-F = 27.266, R^2^ = 0.23, *p* = 0.001; 999 permutations).

The correlation matrix between accumulated metal concentrations, the Metal Pollution Index (MPI), and the normalized fluorescence emission in fish samples is presented in Table 2. A strong positive correlation was found between the MPI and emission at 440 nm (r = 0.7246), driven mainly by Al (r = 0.6702) and Cd (r = 0.5001).

**Figure 3:**
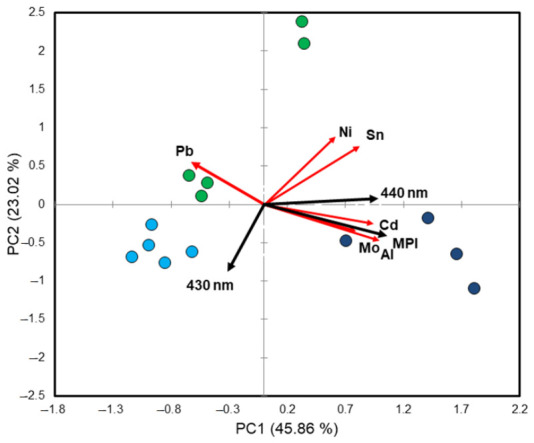
PCA biplot (PC1 vs. PC2) illustrating the discrimination of fish samples from the Pek (dark blue), Ibar (green), and Kruščica (light blue) based on certain metal concentrations, Metal Pollution Index (MPI), and normalized fluorescence emission intensities.

The PCA biplot (Figure 3) explained 68.88% of the total variance (PC1: 45.86%, PC2: 23.02%), indicating a robust representation of the dataset. Fish samples were clearly separated into three geographic clusters corresponding to the Pek, Ibar, and Kruščica sampling locations.

### 3.4. Health Risk Assessment

#### 3.4.1. Target Hazard Quotient (THQ)

**Figure 4:**
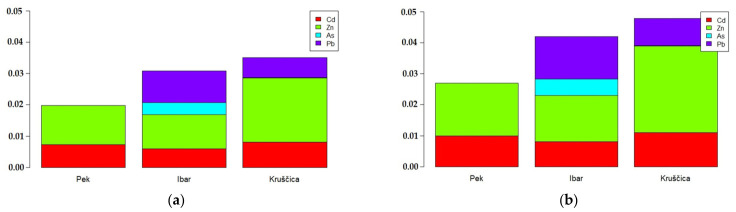
Target Hazard Quotient (THQ) values for (**a**) males and (**b**) females resulting from the consumption of chub from the three investigated localities.

The calculated THQ values for individual elements, as well as TTHQ, were below the established safety threshold (value of 1) for both males and females (Figure 4).

The highest contribution to TTHQ was observed for Zn in both males and females at all sampling sites (Figure 4). For individual fish, the maximum THQ values observed for males were 0.02, 0.08, and 0.08 for the Pek River, Ibar River, and Kruščica Reservoir, respectively. For females, the highest individual THQ values were 0.04, 0.11, and 0.11 for the Pek River, Ibar River, and Kruščica Reservoir, respectively. In the Pek River, only Zn and Cd were detected. As Cu was identified in only one from the Ibar River, it was excluded from further analysis. The THQ for Cr is not presented because its values were considerably lower than those of the other elements.

#### 3.4.2. Target Carcinogenic Risk Factor (TR)

Higher TR values were recorded for Pb compared to As for both males and females at the Kruščica Reservoir, whereas the opposite trend was observed at the Ibar River (Table 3). When comparing locations, lower TR values for both inorganic As and Pb were found at the Kruščica Reservoir compared to the Ibar River (As and Pb concentrations were not detected in the Pek River). At both sites, TR values were higher in females than in males.

## 4. Discussion

### 4.1. Element Analysis

Sample sizes were limited by ethical considerations, collection permits, and practical constraints associated with sampling wild fish populations. Nevertheless, the number of individuals analyzed was comparable to that used in similar ecotoxicological studies involving natural fish populations, e.g., [4,7,14]. Concentrations of elements in the muscle tissue of chub varied among sampling sites. Analysis of 29 elements in chub muscle revealed that 11 elements showed statistically significant differences between locations.

The highest concentrations of certain elements detected in the muscle tissue of individuals from the Kruščica Reservoir indicate a combination of natural influences, including the local geological structure characterized by magmatic rocks and marine-origin carbonate sedimentary complexes enriched in Cu, Fe, Ni, and Sr [19]. In addition, specific environmental conditions within the reservoir likely promote the deposition and retention of these elements [19]. Similar findings emphasizing the strong influence of geological background on element accumulation in European chub have also been reported by Sunjog et al. [4], who demonstrated this pattern across sites with contrasting characteristics, including the protected Zlatar Reservoir (low anthropogenic impact), the drinking water Garaši Reservoir, and the mining-impacted Peštan River. Additionally, these findings have been attributed to physiological acclimation of the fish and differences in fish diet [19].

During the selected sampling periods, Pek River showed higher water mineralization, reflected in elevated conductivity, Mg concentrations, and sulphate levels, as well as higher concentrations of Fe, Mn, and Al [29]. In addition, the Pek River exhibited better oxygenation conditions, with higher dissolved oxygen concentrations and oxygen saturation values. In contrast, the Ibar River was characterized by considerably higher concentrations of Cu, Cd, Co, and Sb, together with extremely elevated chloride concentrations and higher total nitrogen content [29]. Although there are no available water quality data for the Kruščica Reservoir, data exist for the Zaovine Reservoir [16], which receives inflow from the Kruščica system. According to these findings, Zaovine can be characterized as a relatively “clean” reservoir, exhibiting low concentrations of most metals, elevated phosphorus levels compared to the Ibar and Pek Rivers and reduced overall mineralization. It is further classified as oligotrophic to mesotrophic system, with comparatively low levels of metal contamination. Similarly, sediment samples from the Pek River showed extremely high Cu contamination, indicating heavy pollution associated with mining activities [30]. At the same time, sediments from the Ibar River were characterized by elevated concentrations of Zn, Pb, Ni, and Cd, suggesting substantial anthropogenic pressure and considerable ecological risk. In contrast, the Zaovine Reservoir showed a substantially lower level of contamination and ecological risk [31].

The Pek River exhibited the highest level of anthropogenic pressure, as reflected in elevated bacterial contamination (total coliforms and *Escherichia coli* in the water), pronounced oxidative stress responses in the gills and liver of European chub, increased genotoxic damage, and more severe histopathological alterations in fish gills [32]. Conversely, the Kruščica Reservoir showed the lowest intensity of biological responses to stressors and the lowest overall integrated biomarker response (IBRv2) values, indicating the most favourable ecological status among the investigated sites [32].

Across the investigated locations, the European chub did not prove to be a reliable indicator of Mn, Al, As, Cd, Cr, Cu, and Fe contamination—elements frequently of interest in ecotoxicological research [5,33,34]. No statistically significant differences were found among the studied sites regarding the concentrations of Al, As, Cd, and Cr in either the gills or liver of European chub [32]. However, particular attention should be given to the elevated Cd levels recorded in individuals from all three studied sites. Cd exposure poses significant health risks at the cellular level, primarily through oxidative stress and DNA damage. It disrupts the homeostasis of essential elements such as Zn, Mg, and Cu and is associated with a range of adverse effects, including respiratory symptoms, renal failure, bone and neurological disorders, reproductive dysfunction, anosmia, and increased cancer risk [35,36]. The USEPA [37] classified Pb as a Class-B2 carcinogen and it has been reported to cause adverse effects on the nervous, skeletal, reproductive, renal, and cardiovascular systems [38,39]. The presence of isolated exceedances of Cd and Pb concentrations in fish muscle, although not reflected in mean values or overall risk indices, should be interpreted with caution. Given the high toxicity, persistence, and bioaccumulative nature of these elements, even sporadic exceedances may contribute to long-term exposure in human populations consuming fish regularly. Chronic exposure scenarios are particularly relevant for subsistence or recreational fishers, as repeated intake over time may increase the risk of adverse health effects [31]. These findings therefore highlight the importance of considering both average concentrations and distributional variability when evaluating food safety and human health risk.

The highest MPI values for the Pek River can be attributed to the main pollution sources along the river, namely the aforementioned “Majdanpek” copper mine, which discharges wastewater into the Veliki Pek and Mali Pek rivers, as well as a copper pipe factory and the “Kaona” quarry located directly on the Pek River. The “Majdanpek” copper mine is the primary source of potential hazards. The last major incident occurred in 1974, when approximately 10 million m^3^ of tailings were released into the Pek River, contaminating it downstream to its confluence with the Danube River. Previous investigations of the Pek River [30,40] reported repeated episodes of increased turbidity due to elevated concentrations of dissolved solids, as well as particularly high levels of Cu, Fe, Mn, N, sulphates, ammonia, and orthophosphates in both water and sediments. Some studies assessing the impact of mining on aquatic organisms have focused on altered pH values and increased concentrations of metal ions in water and substrate to determine their harmful effects and underlying mechanisms [41]. Although the highest individual element concentrations were recorded in the muscle tissue of fish from the Kruščica Reservoir, the highest MPI values in individuals from the Pek River indicate that anthropogenic pollution plays a dominant role in the overall burden of potentially toxic elements in aquatic ecosystems [42].

### 4.2. Fluorescence Spectroscopy of Muscle Tissue

The emission maxima observed in fish muscle spectra originate from collagen, the principal structural and supportive protein [43], which forms a supporting network that maintains the integrity and mechanical stability of muscle fibres [44]. According to literature data, collagen consisting of longer amino acid chains with preserved inter-chain cross-links exhibits emission maxima at higher wavelengths, whereas hydrolyzed collagen, composed of shorter chains with fewer cross-links, emits at lower wavelengths [45,46]. Given that collagen is a key structural component in muscle tissue, changes in muscle emission spectra can be associated with alterations in collagen integrity. The red shift of collagen emission maxima observed in muscles from the three rivers follows the gradient of pollution burden (Kruščica < Ibar < Pek). This shift towards longer wavelengths suggests an increase in effective chain length or structural rearrangement of collagen, likely driven by oxidative processes induced by environmental pollutants. Similar red shifts have been associated with interactions of organic matter and metal ions with biological fluorophores, leading to stabilization of the excited state and emission at longer wavelengths [47,48]. The fluorescence results are consistent with findings obtained using other analytical approaches in this study, which revealed an elevated metal burden in the Pek River, contributing to structural modifications in muscle tissue. Although the results were associated with differences in elemental burden among sites, these fluorescence spectral shifts cannot be interpreted exclusively as a consequence of pollution pressure. The observed variability likely reflects the combined effects of contaminant exposure, natural environmental variability, physiological condition, and/or tissue biochemical composition. Future multidisciplinary studies combining spectroscopic, histological, and biochemical approaches are needed to validate the biological basis of the observed fluorescence responses.

PCA and correlation analyses revealed a clear relationship between heavy metal accumulation and fluorescence changes in fish muscles. Pek River samples were associated with MPI, Al, Mo, Cd, and the 440 nm fluorescence signal, indicating the highest metal burden, while Ibar samples were characterized by elevated Pb, Ni, and Sn levels reflecting a distinct industrial contamination profile. Kruščica samples were positioned opposite to the metal vectors, consistent with lower contamination. The 430 nm fluorescence vector was oriented opposite to the Sn and Ni vectors, suggesting fluorescence quenching associated with these metals. In contrast, the 440 nm emission was aligned with MPI and major metal pollutants, supporting its role as a biomarker of metal bioaccumulation and ecotoxicological stress. The close alignment of the Sn and Ni vectors confirms their strong positive correlation, indicating a common anthropogenic source. Likewise, the clustering of MPI, Al, and Mo along the positive PC1 axis suggests that Al and Mo are major contributors to the overall metal burden. Their association with Pek samples points to a characteristic mining-related multi-metal contamination pattern.

The strong correlation between MPI and the 440 nm emission peak, together with the alignment of the 440 nm vector with major pollutants, supports the use of this wavelength as a biomarker of cumulative ecotoxicological stress. The enhanced 440 nm emission may be related to metal-induced alterations of structural proteins, including collagen [49,50], and the accumulation of endogenous fluorophores such as NADH [51]. In contrast, the opposite orientation of the 430 nm vector relative to Sn and Ni suggests fluorescence quenching associated with these metals. The strong Sn–Ni correlation further indicates a common anthropogenic source of contamination.

### 4.3. Health Risk Assessment

Regarding long-term exposure to non-carcinogenic pollutants, the consumption of European chub meat can be considered safe for human nutrition. Interestingly, the lowest TTHQ values were recorded for the Pek River, primarily because As and Pb concentrations in individuals from this river were below the detection limits. Slightly higher TTHQ values were observed for women than for men, which can be attributed to lower average body weight and longer life expectancy in women [52,53].

The TR for the Pek River was not estimated because As and Pb concentrations were below the detection limit. The cancer risk associated with consumption of European chub from the Kruščica Reservoir and the Ibar River with respect to Pb and As can be considered negligible, while for As at the Ibar River it falls within the tolerable range. In this case as well, gender-related exposure factors resulted in relatively higher TR values for women than for men. Similar results were obtained for European chub from five reservoirs with different characteristics and purposes in Serbia, where the reservoir used for water supply was identified as the safest site in terms of fish consumption [3].

Although Pb and Cd exceeded the MAC values in some individuals, these exceedances did not translate into THQ and TR values above the safety threshold. The health risk assessment presented in this study is subject to uncertainty because it relies on several assumptions, including average fish consumption rate, body weight, life expectancy, and the assumption that inorganic As represents 3% of total As. Therefore, the calculated THQ and TR values should be considered screening-level estimates rather than precise measures of individual risk. Additionally, individuals with higher fish consumption rates (i.e., recreational and commercial fishermen) may experience greater exposure than estimated using average consumption scenarios.

## 5. Conclusions

Fish have the capacity to accumulate toxic elements within their tissues while maintaining physiological homeostasis, enabling normal functioning and survival. Through adaptive mechanisms, they can tolerate chronic exposure to elevated, yet non-lethal, concentrations of these elements in their environment, whether the contamination arises from natural processes or anthropogenic activities. Conversely, the analysis of biomarkers—such as fluorescence-based assays—can reveal morpho-physiological responses indicative of exposure to environmental pollutants. The muscle tissue of the European chub did not show significant differences in the accumulation of most elements, regardless of sampling site or degree of pollution exposure. This pattern likely reflects its relatively low affinity for element accumulation and/or effective physiological regulation of these elements in this tissue type. The interpretation of site-specific differences should take into account the relatively limited sample size and the high natural variability characteristic of wild fish populations. Nevertheless, fluorescence analysis indicated potential subtle structural or biochemical variations that may reflect organism-level responses to differing environmental conditions. Specific fluorescence emission is a robust optical indicator of pollution severity (MPI) and distinct heavy-metal stressors. This study suggests that, in the practical assessment of pollution burden in fish muscles, results obtained from rapid, non-destructive fluorescence spectroscopy can be effectively used as a preliminary screening tool, enabling the identification of samples requiring further examination with complementary, more sensitive, and precise analytical methods.

## Figures and Tables

**Table 1:** Number of individuals (n), total length (TL, cm), weight (W, g), metal pollution index (MPI), and element concentrations (μg/g dw) in muscle of chub sampled in three aquatic ecosystems in Serbia. Values are presented as mean ± SD. ND stands for values below the detection limit (0.0198, 0.0325, 0.0205, 0,00471, and 0.008667 for As, Cu, Pb, Ag, and Sb, respectively).

	Pek	Ibar	Kruščica
*n*	10	10	8
TL	14.20 ± 1.63	17.81 ± 5.82	15.11 ± 3.62
(min–max)	12.8–18.2	13.1–29.5	11.7–21.8
W	24.70 ± 9.35	75.50 ± 89.70	35.13 ± 29.22
(min–max)	18–49	17–140	13–91
MPI *	0.044 ± 0.013 ^b^	0.025 ± 0.013 ^a^	0.024 ± 0.011 ^a^
Al	1.50 ± 2.43	0.87 †	0.31 †
As	ND	0.17 ± 0.22	0.03 †
Ba *	0.48 ± 0.13 ^b^	0.21 ± 0.16 ^a^	0.41 ± 0.20 ^b^
Cd	0.03 ± 0.03	0.03 ± 0.04	0.03 ± 0.06
Cr	0.05 ± 0.02	0.06 ± 0.15	0.04 ± 0.05
Cu	ND	0.13 †	ND
Pb **	ND ^a^	0.17 ± 0.19 ^a^	0.11 ± 0.13 ^b^
Se **	1.26 ± 0.23 ^a^	0.27 ± 0.24 ^ab^	0.37 ± 0.37 ^b^
Ag	0.95 †	ND	ND
Sb **	ND ^a^	0.10 ± 0.14 ^a^	0.08 ± 0.11 ^ab^
Mo **	0.02 ± 0.02 ^b^	0.041, 0.045 ‡^ab^	ND ^a^
Pt	0.01 ± 0.02	0.07 ± 0.08	0.16 ± 0.19
Sn **	0.07 ± 0.03 ^b^	0.05 ± 0.05 ^ab^	0.03 †^a^
Ti	0.04 ± 0.08	0.05 ± 0.05	0.162, 0.072 ‡
Sr **	5.71 ± 2.34 ^b^	1.11 ± 1.15 ^a^	6.78 ± 5.77 ^b^
Fe	5.24 ± 1.23	6.19 ± 3.22	4.30 ± 1.23
Zn **	16.12 ± 2.32 ^a^	14.12 ± 7.17 ^a^	26.39 ± 6.74 ^b^
Ca **	1405.72 ± 554.81 ^b^	653.94 ± 370.50 ^a^	2055.95 ± 1574.28 ^b^
Mg	322.71 ± 46.03	315.47 ± 48.49	358.69 ± 42.70
P **	2943.63 ± 1171.91 ^a^	3155.75 ± 438.01 ^a^	4261.14 ± 1156.44 ^b^
K *	3889.67 ± 524.11 ^a^	4300.87 ± 429.42 ^a^	4402.19 ± 216.14 ^b^
Na *	353.84 ± 112.76 ^a^	357.63 ± 154.73 ^a^	594.20 ± 132.71 ^b^
S **	2051.87 ± 602.73 ^a^	2713.83 ± 441.79 ^b^	2305.34 ± 166.13 ^ab^
Ni **	0.08 ± 0.02 ^a^	0.09 ± 0.07 ^a^	0.11 ± 0.31 ^b^

^a.b^ Values with different letters in the same row are significantly different (Mann–Whitney *U*-test. *p* ≤ 0.05 or Tukey HSD *post hoc* test. *p* ≤ 0.05). * Significant differences between sampling locations (Kruskal–Wallis *H*-test. *p* ≤ 0.05). ** Significant differences between sampling locations (one-way ANOVA *p* ≤ 0.05). † Concentrations above the detection limit in a single sample only. ‡ Concentrations above the detection limit in two samples only.

**Table 2:** Pearson correlation matrix between certain metal concentrations, Metal Pollution Index (MPI), and normalized fluorescence emission in fish samples.

	Al	Cd	Pb	Mo	Sn	Ni	415 nm	430 nm	440 nm	MPI
Al	**1**									
Cd	**0.7040**	**1**								
Pb	**−0.5457**	**−0.5935**	**1**							
Mo	**0.7210**	0.4484	−0.2062	**1**						
Sn	0.3316	0.4126	−0.0104	0.3230	**1**					
Ni	0.1248	0.2954	0.2033	0.2268	**0.9519**	**1**				
415 nm	−0.2498	−0.1248	0.1964	−0.0404	−0.3207	−0.2417	**1**			
430 nm	0.0826	−0.1569	0.0964	0.1180	**−0.5740**	**−0.5568**	0.4761	**1**		
440 nm	**0.6702**	0.5001	−0.3035	0.4371	**0.5957**	0.4286	**−0.6071**	−0.0635	**1**	
MPI	**0.8205**	**0.7712**	**−0.6422**	**0.6952**	0.4099	0.2289	−0.2386	0.0407	**0.7246**	**1**

Values in bold are different from 0 with a significance level α = 0.05.

**Table 3:** Target cancer risk (TR) values for Pb and As in males and females associated with the consumption of chub sampled from the Kruščica reservoir and the Ibar River. TR values for the Pek River are not presented because Pb and As concentrations at this site were below the detection limit (ND).

		Pb		As	
		Males	Females	Males	Females
Kruščica	mean	2.2 × 10^−7^	3.0 × 10^−7^	5.3 × 10^−8^	7.3 × 10^−8^
	max	6.4 × 10^−7^	8.8 × 10^−7^	3.2 × 10^−7^	4.4 × 10^−7^
Ibar	mean	3.4 × 10^−7^	4.7 × 10^−7^	1.7 × 10^−6^	2.4 × 10^−6^
	max	7.0 × 10^−7^	9.6 × 10^−7^	7.0 × 10^−6^	9.6 × 10^−6^

## Data Availability

The original contributions presented in this study are included in the article/Supplementary Material. Further inquiries can be directed to the corresponding author.

## Supplementary Materials

The following supporting information can be downloaded at: https://www.mdpi.com/article/10.3390/toxics14070580/s1, Table S1: Limits of detection—LOD (mg/L), limits of quantification—LOQ (mg/L), and recovery values (%) for analyzed elements.
