# Supplementary material for: From Environmental Burden to Structural Alterations: Integrating Chemical Analysis and Fluorescence Spectroscopy in European Chub (Squalius cephalus)
Source: Toxics. 2026 Jun 30;14(7):580. doi: 10.3390/toxics14070580 (PMC13416709; doi:10.3390/toxics14070580)
Supplement: Supplementary file 1 [file toxics-14-00580-s001.zip › toxics-4330136-supplementary.pdf]

Table S1. Limits of detection – LOD (mg/L), limits of quantification – LOQ (mg/L), and recovery values (%) for analyzed elements.

	LOD	LOQ	Recovery value
Al	0.01	0.025	90-110
As	0.005	0.025	90-115
Ba	0.01	0.025	90-110
Cd	0.01	0.025	90-110
Cr	0.005	0.025	90-110
Cu	0.005	0.01	90-110
Pb	0.01	0.05	90-110
Se	0.02	0.05	90-115
Ag	0.01	0.025	90-110
Sb	0.01	0.025	90-110
Mo	0.005	0.015	90-110
Pt	0.01	0.015	90-110
Sn	0.01	0.025	90-110
Ti	0.005	0.025	90-110
Sr	0.001	0.0025	90-110
Fe	0.001	0.01	90-110
Zn	0.005	0.01	90-115
Ca	0.005	0.5	90-110
Mg	0.001	0.5	90-110
P	0.01	0.5	90-110
K	0.01	1.5	90-115
Na	0.005	0.5	90-110
S	0.5	1.5	90-110
Ni	0.01	0.025	90-115
